# The Changing Concepts of the Constitution

**DOI:** 10.1093/ojls/gqac001

**Published:** 2022-01-19

**Authors:** Alex Schwartz

**Keywords:** British constitution, constitutional change, unwritten constitution, UK constitution, word embedding, word2vec

## Abstract

There have been several important formal changes to the United Kingdom’s constitution over the past few decades, including devolution to Northern Ireland, Scotland, and Wales; the incorporation of the European Convention on Human Rights in domestic law; and the creation of a new Supreme Court. This article is about the informal semantic changes that may have accompanied these formal changes. It focuses on several central concepts: parliamentary sovereignty, the rule of law, the separation of powers, devolution, and human rights. Using a recently developed machine learning method to analyse a massive corpus of parliamentary debate, the article gauges the extent to which these concepts have become more (or less) related to the meaning of the UK’s constitution in parliamentary discourse. Ultimately, the analysis supports some important theoretical expectations about the changing nature of the constitution, including the claim that parliamentary sovereignty is now a less significant concept for the meaning of the constitution than it once was.

## 1. Introduction

According to a prevailing view in public law scholarship, the last two decades have been an extraordinary period of constitutional change for the UK.^1^ And there is good reason for this appraisal. In the late 1990s, Tony Blair’s ‘New Labour’ government launched a series of reforms that rearranged much of how public power in the UK is allocated and exercised: legislative competencies were devolved to new substate assemblies in Northern Ireland, Scotland and Wales;^2^ the European Convention on Human Rights was made enforceable in domestic courts;^3^ the hereditary element within the legislature was at least partly reformed;^4^ judicial appointments were modernised and insulated from executive influence;^5^ and a new apex court was created to replace the Appellate Committee of the House of Lords.^6^ So ambitious were these reforms that they might even be said to have created a *new* constitution.^7^ Moreover, the ensuing years have made Blair’s project look like it may have been only the first phase of an indefinite state of flux in the UK’s constitutional affairs^8^—a state of ‘constitutional unsettlement’.^9^ Every successive government since Blair’s government has implemented (or attempted to implement) changes of a broadly constitutional nature,^10^ the most dramatic of these being the UK’s recent withdrawal from the European Union.^11^

We should not be surprised to discover that the UK’s constitution has changed or is still changing. From a comparative perspective, constitutional change is a regular and even banal event.^12^ That being said, the question of constitutional change in the UK has some idiosyncratic features. Unlike nearly all other contemporary states, the UK lacks a definitive and entrenched constitutional text. Some statutes—such as the Human Rights Act 1998 or the Constitutional Reform Act 2005—perform functions that are probably best understood as ‘constitutional’, in the sense that they organise or limit public power, but these ‘constitutional statutes’ are not formally entrenched against ordinary legislative repeal or amendment.^13^ Moreover, much of what people talk about when they talk about the UK’s constitution are its informal elements—the ‘political constitution’ comprising ‘a set of relatively “thick” and deeply embedded normative values and principles which provide how state power should be exercised and constrained’.^14^ In shaping notions of constitutional propriety, ‘tacit understandings’^15^ about these values and principles are thought to play a critical role in the discursive practice of contesting and exercising public power.^16^ But because these understandings are not codified in any canonical way, this aspect of the constitution may change as people—specifically, political elites in Parliament and in government—begin to use (and perhaps also understand) the relevant concepts in a different way. In other words, a change in discourse may occasion a semantic shift in the conceptual scheme of the UK’s constitution.^17^ Relative to the formal changes that might be enacted by constitutional statutes, informal semantic changes are bound to be more difficult to detect, track and evaluate.^18^

This article takes up the challenge of investigating semantic changes in the conceptual scheme of the UK’s constitution, at least in so far as those changes might be reflected in parliamentary debate. My primary aim here is to gauge the extent to which several concepts—parliamentary sovereignty, devolution, the rule of law, the separation of powers and human rights—have become more (or less) related to the meaning of the UK’s constitution over the past few decades. To this end, I enlist the help of a recent innovation in computational methods—a machine learning algorithm, colloquially known as ‘Word2Vec’—to analyse a massive corpus of parliamentary debate texts. This novel approach allows theoretical expectations about semantic change to be tested empirically. Among other things, the findings presented here support the still-controversial claim that parliamentary sovereignty has become a less significant concept for the meaning of the UK’s constitution than it once was. The article’s secondary aim is to demonstrate the burgeoning power of new computational methods for legal research more generally. Although the computational method used here (ie word embedding) has been used by other fields—linguistics, sociology, political science and the digital humanities—this article is the first instance of it being used to inform UK public law scholarship.^19^ Beyond the field of public law, this method can be adapted to the study of discourse about any legal or quasi-legal phenomenon. Legal scholars, particularly those who are interested in analysing massive textual sources, will find this method to be a distinct and valuable complement to more familiar approaches. With this general audience in mind, the article provides a step-by-step explanation of its methodology.

The article is structured as follows. In section 2, I review the academic literature and elicit several theoretical expectations about semantic change in the conceptual scheme of the UK’s constitution. In section 3, I explain how machine learning is used to model semantic relations between concepts; I canvass the various methodological issues involved in gathering, processing and organising the relevant textual data; and I discuss how estimates of semantic change were created for the purposes of the ensuing analysis. In section 4, I analyse these estimates of semantic change to determine which, if any, theoretical expectations are vindicated. In section 5, I conclude with a discussion of the implications and limitations of the article’s findings.

## 2. Theorising the Changing Concepts of the Constitution

As is typical of constitutions in general, it is difficult (and perhaps even impossible) to detach positive description of the UK’s constitution from normative evaluation. Certain constitutional principles and conventions loom larger than others in popular and scholarly imaginations, but people disagree about the meaning and relative priority of even the more salient principles and conventions. AV Dicey’s account of parliamentary sovereignty—that Parliament may ‘make or unmake any law whatever; and, further, that no person or body is recognised by the law … as having a right to override or set aside the legislation of Parliament’^20^—still exerts a kind of gravitational pull on British constitutional thought,^21^ but this old orthodoxy is now widely and predictably contested.^22^ Commentators also disagree about whether (or to what extent) putative aspects of the constitution may have changed over the past few decades. Indeed, there is a range of plausible perspectives about the nature and extent of recent constitutional change in the UK.

Vernon Bogdanor is perhaps the most forceful proponent of the claim that New Labour’s reforms radically transformed the UK’s constitution.^23^ Bogdanor contends that those reforms ‘crucially and, almost certainly, permanently undermined’ traditional assumptions about the constitution, particularly assumptions relating to parliamentary sovereignty.^24^ Of course, the UK’s membership in the European Union (and before that, the European Community) had already limited Parliament’s ability to legislate, and domestic courts would disapply legislation found to be inconsistent with European law.^25^ However, according to Bogdanor, New Labour’s reforms created a sharper and more systematic discontinuity with the past, supplanting the ‘traditional’ constitution—a constitution based primarily on the principle of parliamentary sovereignty—with a ‘new’ constitution that institutionalises a formal separation of powers.^26^ This new constitution, he says, is more a product of deliberate design and is more reliant on statutory form;^27^ it is quasi-federal (because of devolution to Northern Ireland, Scotland and Wales);^28^ and it provides for a judicially enforceable catalogue of fundamental rights—something ‘very near to a bill of rights’—in the Human Rights Act 1998.^29^ As Bogdanor puts it, ‘the constitution so brilliantly analysed by Bagehot and Dicey no longer exists’.^30^

Bogdanor is not alone in suggesting the decline of parliamentary sovereignty and a more juridical constitution.^31^ In fact, as many readers will recall, some senior members of the judiciary have suggested more or less the same idea, albeit in obiter dicta.^32^ But not all commentators have agreed that New Labour’s reforms really did achieve such a profound constitutional revolution. Writing in 2008, Aileen McHarg observed that claims about the juridification of the constitution seemed to underestimate the extent to which the UK’s governing elites are committed to a ‘political constitutionalism’ guided by flexible conventions and other forms of ‘soft law’.^33^ Others have questioned the extent to which the Human Rights Act 1998 really is in tension with an orthodox account of parliamentary sovereignty and political constitutionalism. In her 2008 book, Alison Young argued that the Human Rights Act is perfectly consistent with a Diceyan account of parliamentary sovereignty (properly understood).^34^ And Richard Bellamy, writing in 2011, argued that the Human Rights Act might have strengthened the political constitution, noting how the Act facilitates greater parliamentary scrutiny of prospective legislation for compliance with human rights while also providing the courts with a post-legislative mechanism—the ‘declaration of incompatibility’—to prompt further parliamentary deliberation about the proper scope and protection of human rights.^35^

But the question of constitutional change need not be framed as a binary contest in which a new legal constitution does or does not supplant the old political constitution. Neil Walker suggests that we might do better to understand the UK’s constitution as having entered an indefinite state of ‘unsettlement’—that is, a phase in which various questions about the fundamental form and substance of the constitution ‘are subject to continuous disputation with deeply uncertain long-term consequences, regardless of how they may be resolved in the present tense’.^36^

Thinking about constitutional change in terms of evolving discursive practice (as opposed to definitive settlement) is productive for at least two reasons. For one thing, it allows us to sidestep some vexing questions about the ‘correct’ interpretation of recent constitutional reforms, questions which may well be intractable unless and until there is a head-on collision between the courts and Parliament that is intense enough to test ‘a different hypothesis of constitutionalism’.^37^ Moreover, in so far as the UK’s constitution is still highly reliant on an informal conceptual scheme of principles and values, changes in how political elites speak about the relevant concepts may have practical implications for how the boundaries of constitutional propriety are understood and enforced. Thus, fixing our attention on discursive change may reveal subtle but important dimensions of informal constitutional change.

One way in which a change in discourse may matter is that it produces a change in the *meaning* of key constitutional concepts. Now it is important to clarify what is meant here by ‘meaning’. In one sense, the meaning of a concept might be equated with a kind of timeless truth: an *a priori* idea with an essential content that is independent of how people at any given time and place understand it. In this *ahistorical* sense of meaning, there can be a correct interpretation of even a highly contested concept—‘the rule of law’, for example—and it is even likely that most people have been mistaken about what the concept truly means.^38^ But another way to think about the meaning of a concept is to see it as a dynamic social construction that is largely (if not entirely) contingent on changeable patterns in the way words are used.^39^ In this sense, the meaning of concepts cannot be fixed independently of how those concepts (or, more specifically, the words used to express them) feature in the discourse of particular communities in particular times and places.^40^ As a concept is deployed in different contexts and for different purposes, it may acquire new patterns of use and, ultimately, its ‘meaning’ may shift.^41^ A change in a community’s discourse is therefore a window of opportunity for semantic change.^42^

The field of historical linguistics has identified various types of lexical semantic change.^43^ The classical types involve a change in a word’s denotation through a broadening or narrowing of reference or, alternatively, through the convergence of one word’s denotation with that of another.^44^ A familiar example of lexical semantic change is what happened to the word ‘tea’ in certain dialects of British English over the course of the 19th century: as practices adapted to changing prices of goods, the word ‘tea’ (sometimes also ‘high tea’) came to denote not just the hot beverage, but also a culturally specific concept of an evening meal.^45^ More subtle semantic shifts relating to a word’s connotations have also been observed: words can become more or less *semantically related* to one another within some particular discursive domain.^46^ For example, the words ‘turkey’ and ‘goose’ were once commonly used in the same sort of relation to ‘Christmas dinner’. In virtue of this common relationship, these words were semantically related within what we might call (only slightly tongue-in-cheek) ‘the discourse of Christmas’. However, the relevant practices have changed over the last 200 years (people eat less goose and much more turkey at Christmas) so that ‘turkey’ can be said to have become much more semantically related to the concept of ‘Christmas dinner’ than its gamier cousin. These gastronomical examples may seem trite, but the notion that subtle kinds of semantic change can be prompted by changes in discursive practice is important and illuminating for present purposes—it gives us a way to think about how changes in constitutional discourse might occasion semantic change across a range of concepts associated with the UK’s constitution.

At the most general level, there are reasons to think that the meaning of the concept of the constitution itself may have changed. Martin Loughlin suggests something along these lines.^47^ He proposes that one consequence of New Labour’s reforms is that, for the first time in the British context, the dominant meaning of ‘constitution’ has been aligned with the modern template of ‘constitution’ as a ‘normative framework protected by law … laying down the terms of the compact between citizens and government’.^48^ Signs of this broadly more juridical sense of ‘constitution’ include the ascendency of the theory of ‘constitutional statutes’, which suggests a more codified (less unwritten) understanding of constitutional form,^49^ as well as the emergence of a new theory of ‘common law constitutionalism’, suggesting an unwritten but nonetheless justiciable core of ‘constitutional’ rights and liberties.^50^

One can go beyond these general intimations of conceptual change to specify more precise expectations, namely that certain concepts may have become more (or less) semantically related to the concept of the constitution since New Labour’s reforms. In other words, some concepts may have gained (or lost) some grade of *constitutional resonance*. The intuition behind such expectations is that the concept of the UK’s constitution is not a monolithic whole, but rather a complex and dynamic constellation of ideas. A concept’s ‘constitutional resonance’, then, is the extent to which that concept’s meaning is bound up with (and thus contributes to) the meaning of the broader concept of the constitution within some relevant discourse. This intuition implies that constitutional resonance will vary from one concept to another—some concepts may be so semantically related to the constitution that they are practically synonymous with it; others may have only a marginal constitutional resonance; still others will have no constitutional resonance whatsoever. This intuition also implies that a concept’s constitutional resonance may vary over time, as the discourse associated with that concept becomes more or less convergent with discourse about the constitution. Thus, a concept’s constitutional resonance at any given time should not be taken to imply anything about that concept’s semantic stability. Nor should constitutional resonance be taken to imply a consensus about a concept’s meaning; a concept that is very closely associated with the constitution may nevertheless be a hotly contested one. In the context of the United States, for example, ‘freedom of speech’ is presumably a concept with a very high degree of constitutional resonance. People fervently disagree about the scope and implications of freedom of speech, but the language that is used to express this disagreement will nevertheless reflect a close semantic relationship to the US constitution. Likewise, in the context of the UK, we should expect that some concepts of great constitutional resonance will nevertheless be the subject of considerable disagreement. Indeed, disagreement and constitutional resonance may often go hand in hand; it may be precisely because a concept has acquired a heightened constitutional resonance that it becomes more contested. Alternatively, we should not rule out the possibility that contestation about the meaning of a concept may prompt a change in that concept’s constitutional resonance.

Why might a change in a concept’s constitutional resonance matter? Presumably, semantic change of this kind will have implications for the trajectory of constitutional change in the UK, particularly with respect to the more informal dimensions of the constitution. If we assume, as many scholars have argued, that the UK’s constitution is at least partly a function of the meanings that certain actors assign to certain concepts in discursive practice,^51^ then changes in the constitutional resonance of those concepts may be signs of informal constitutional change. As a concept acquires greater constitutional resonance—that is, as its meaning becomes more bound up with the meaning of the constitution—we would expect it to play a weightier role in contesting or determining prevailing notions of constitutional propriety. Conversely, as a concept loses constitutional resonance, its role with respect to notions of constitutional propriety may be diminished.

With this understanding of constitutional resonance in mind, several plausible hypotheses follow. The first relates to the concept of parliamentary sovereignty. Notwithstanding the surviving influence of the old Diceyan doctrine, the way in which the concept of parliamentary sovereignty features in contemporary discourse cannot help but be ‘shaped by the changing nature of the constitutional landscape in which it sits’.^52^ As we have seen, this ‘constitutional landscape’ is a much more complex terrain than it once was; reasons to equivocate about parliamentary sovereignty abound. To be sure, one may still claim that parliamentary sovereignty is the ‘dominant characteristic’^53^ of the UK’s political institutions, but this claim will be accompanied by caveats and significant qualifications, and, within the parameters of the very same discourse, one’s interlocuter might plausibly respond that ‘[i]n practice, if not in law, parliamentary sovereignty is no longer the governing principle of the British constitution’.^54^ There are also broader socio-political changes at work that may have weakened the constitutional resonance of parliamentary sovereignty. As Loughlin and Stephen Tierney suggest, a range of factors—a decline in public trust of elected representatives, an increased reliance on secondary legislation and referendums, and a general ‘disaggregation of the cultural-political notion of the “British people”’—have rendered Parliament less able to ‘present itself as the authoritative voice of the political nation’.^55^ In sum, there are several reasons to think that the concept of parliamentary sovereignty may now have a weaker constitutional resonance than it once enjoyed.

Other concepts may have acquired *greater* constitutional resonance over the same period of time. The separation of powers is one such concept. Historically, the notion of the separation of powers has been subject to widely divergent accounts and—given the close institutional links that have traditionally existed between the UK’s legislature, executive and judiciary—the concept’s relevance for the UK’s constitution has often been questioned.^56^ But the Constitutional Reform Act 2005 is a deliberate attempt to institutionalise a particular conception of the separation of powers for the first time in the UK’s history.^57^ In the ensuing years, the separation of powers seems to have ‘(re)surfaced as an instrument of constitutional argumentation providing a facilitating framework for constitutional debate and is increasingly invoked in support of decision-making in the politico-legislative arena’.^58^

There are also reasons to think that the concept of the rule of law may have acquired a new and heightened constitutional resonance. Although the principle of the rule of law was recognised by Dicey in the late 19th century as a ‘pillar’ of the UK’s constitution, talk of the rule of law seems to have been emboldened and disseminated across diverse contexts over the last few decades: the rule of law is now recognised explicitly in statute^59^ and by the courts^60^ as a ‘constitutional principle’, and it is used to contest everything from cuts to legal aid to counterterrorism policy.^61^ Furthermore, the conception of the rule of law in play across these various contexts is apparently a polysemous one, broader and more substantive than the relatively austere and formal conception of it that Dicey articulated so long ago.^62^

The foregoing hypotheses about semantic change relate to how old, familiar ideas find new modes of expression in contemporary contexts. But there is a more dramatic and obvious way in which recent reforms may have caused some concepts to acquire a greater degree of constitutional resonance—that is, by taking what were historically peripheral concepts for the British constitutional tradition and writing them into the fabric of contemporary public law. Consider the concept of ‘devolution’. As a term of art in UK constitutional discourse, ‘devolution’ is prefigured by the similar ‘home rule’; both terms have been used, sometimes synonymously, to refer to the delegation of legislative powers to territorial units associated with strong and historically rooted substate national identities.^63^ ‘Home rule’ is still sometimes used to denote an alternative model for Scotland’s place within the UK.^64^ But ‘devolution’ is now the dominant term for talking about the territorial dimensions of the UK’s constitution.^65^ In part, this is an inventible result of the devolution statutes of the late 1990s, which created a system of territorial substate legislatures and a common legal vocabulary (eg ‘devolution issues’).^66^ It is not just that the preferred terminology has changed. The heightened constitutional resonance of devolution is also suggested by the emergence of a related constitutional convention, the Sewel Convention, pursuant to which Parliament will not ‘normally’ legislate in relation to a devolved matter without a prior motion of legislative consent from the relevant substate legislature. This convention was later ‘recognised’ in the Scotland Act 2016^67^ and Wales Act 2017^68^ (though the legal enforceability of the convention was ultimately rejected by the UK Supreme Court).^69^ Moreover, the idea that devolution shall be a ‘permanent’ part of the UK’s ‘constitutional arrangements’ has been given explicit statutory expression (although with uncertain legal consequences).^70^

A similar hypothesis can be formulated in relation to ‘human rights’. It is not as though there was no domestic discourse about individual rights prior to the Human Rights Act 1998—the UK has been party to the European Convention on Human Rights since the 1950s, and has long been home to older discourses of ‘civil liberties’ and ‘natural rights’.^71^ But the Human Rights Act opened up new channels for the modern and more expansive language of human rights to flow into the mainstream of British constitutional discourse.^72^ On one level, the Human Rights Act expands the parameters of legal argumentation to include human rights. Although not an entrenched bill of rights superior in law to other Acts of Parliament, the Human Rights Act gives courts a basis in domestic law for using human rights (specifically, those rights protected by the European Convention on Human Rights) as a ground of judicial review and statutory interpretation.^73^ Furthermore, because courts are themselves public authorities within the meaning of the Human Rights Act, judicial decisions in general (including in private law matters) must be consistent with Convention rights.^74^ The Human Rights Act also creates an entirely new kind of parliamentary speech act: the ministerial ‘statement of compatibility’.^75^ And, to work in tandem with the Act, a new venue for discourse about human rights was created: a permanent joint parliamentary select committee, the Joint Committee on Human Rights, with a mandate to propose legislative changes and draft remedial orders to bring laws into compliance with Convention rights.^76^

The foregoing discussion provides *prima facie* reasons to expect that changes in discursive practice may have prompted semantic changes in the constitutional resonance of certain concepts. These expectations are hypotheses—educated guesses, if you like—informed partly by public law scholarship. But the legal academy is its own special discourse community, with ideas and perceptions that are probably only partially congruent with the dominant understandings of other groups—parliamentarians, government ministers, civil servants and the judiciary—who are more directly involved in the day-to-day practice of the UK’s constitution.

To be sure, one can find anecdotes of judges and Members of Parliament speaking or writing about the UK’s constitution in ways that would seem to support the hypotheses in question. On occasion, judges and parliamentarians are even explicit about their theory of constitutional change. For example, speaking in the House of Lords in December 2002, Liberal Democrat Lord Goodhart expressed a view of the constitution that chimes well with the foregoing theoretical expectations:

The constitution is now very different from the days when I studied constitutional history and constitutional law at school and university some 50 years ago. In those days, the core of teaching about the constitution was still Dicey’s view that there was only one principle of the British constitution—the absolute sovereignty of the Queen in Parliament. Is that still true? I believe that it is not. The European Communities Act, the Scotland Act and the Human Rights Act all restrict the powers of Parliament to legislate. In theory, each of them could be repealed; in practice, I believe that each of them is entrenched. So the constitution today is perhaps a good deal more uncertain than it was in Dicey’s day.^77^

Self-conscious reflections on constitutional change like this may be suggestive, but they are not necessarily representative of elite discourse about the constitution in general. The significance of all of the concepts discussed above—parliamentary sovereignty, the rule of law, the separation of powers, devolution and human rights—is contested. Individual anecdotes can illuminate how particular individuals may understand these concepts, but they cannot provide a general test of the hypotheses in question. To generalise with any confidence about how the concepts of the constitution have (or have not) undergone semantic change within elite discourse, a systematic empirical examination of that discourse is required. In what follows, I explain how this can be done.

## 3. Methodology

### A. Word Embeddings in Semantic Space

Advances in computational methods have opened up new possibilities for the analysis of massive textual corpora. These methods are often grouped together under the banner of ‘natural language processing’ (NLP), and they encompass a range of tools, including relatively simple descriptive statistics of word frequencies and collocation, sentiment analysis and, more recently, machine learning algorithms for modelling substantive topics and other semantic properties in text or discourse. Predictably, scholars from various disciplines have adapted and developed NLP for their respective purposes—the still-burgeoning field of the digital humanities, for example, is defined in large part by the application of computational methods to ‘humanistic’ materials.^78^ NLP methods have even started to find their way into the legal academy with the emergence of computational legal studies.^79^ In each of these areas, NLP has allowed scholars to pose new questions or enhance their ability to address old ones.

One of the new computational tools that can be applied to the study of discourse is ‘word embedding’. Word embedding is a computational method for modelling semantic relationships between words. To do this, each unique word in a corpus of text is assigned its own vector—an *n*-dimensional sequence of numbers—that locates the word within a multidimensional mathematical space. If a word-embedding model is a good one, words with relatively similar or related meanings will be embedded relatively ‘closer’ to one another within this space. In effect, word embedding creates a kind of semantic map, with each word’s vector providing a set of coordinates such that geometric relationships between vectors correspond to semantic relationships between words. Thus, the angle that is formed between two vectors in the embedding space can be used to compute a measure—‘cosine similarity’—of how semantically related or similar one word is to another.

The notion that words might be represented as vectors had been around for some time, but word embedding gained traction for practical and research purposes in 2013, when Mikolov and others (a research team at Google) developed and published a more computationally efficient approach.^80^ The Mikolov and others word-embedding algorithms collectively go under the name ‘Word2Vec’. In common with other approaches to word embedding, the animating intuition of Word2Vec is what linguists call the ‘distributional hypothesis’, ie that words with relatively similar or related meanings will tend to appear in relatively similar linguistic contexts.^81^ Thus, the Word2Vec algorithms train a model to predict a target word from a surrounding window of context words or, alternatively, to predict a surrounding window of context words from a single target word.^82^ In and of itself, this prediction task is not really of much interest (at least, not for present purposes). However, in the process of iterating through the sentences of a corpus to optimise a model for this prediction task, the algorithms generate *n*-dimensional word vectors that will, in theory, encode information about the meaning of the words therein.

This approach allows for two words to have varying degrees of semantic similarity or relatedness with one another, as measured by cosine similarity, reflecting the extent to which those words tend to appear in some similar linguistic contexts but not others. Importantly, the cosine similarity score for two word vectors does not simply reflect the frequency with which the associated words appear together in the same sentences (although this would have some influence on the model). Rather, it is a measure of semantic similarity based on the probability of those words appearing in similar immediate contexts, independent of how often they appear together within the same sentences. Furthermore, because the resultant vectors are multidimensional (the standard approach now is to train word vectors with 300 dimensions),^83^ the Word2Vec algorithms can encode multiple aspects of a word’s meaning.^84^ For example, a Word2Vec model can generate word vectors that reflect both the semantic similarity between ‘cats’ and ‘dogs’ on some dimensions (eg both words refer to household pets) as well as their semantic differences on other dimensions (eg cats are a type of feline and dogs are not). Indeed, the Word2Vec algorithms can do a remarkably good job of encoding even rather subtle semantic relationships. For example, given a large enough training corpus, the vector for the word ‘queen’ will capture a semantic relation to monarchy as well as the word’s gendered meaning. Thus, one can query the model to find that ‘man’ is to ‘king’ as ‘woman’ is to ‘queen’ simply by subtracting the vector for ‘man’ from the vector for ‘king’ and then adding the vector for ‘woman’; if the model has been given sufficient training material, this vector arithmetic should yield the nearest point in the embedding space to the vector for ‘queen’.^85^

The possibility of using word embedding for the study of semantic change has not gone unnoticed. A handful of scholars from various disciplines have already applied the method to investigate, for example, the changing meanings of equality in American news media;^86^ the changing cultural connotations connected to social class as expressed in the text of English language books published over the course of the 20th century;^87^ and changes in the parliamentary discourse on the punishment of war criminals in the decades following the Second World War.^88^ In the same vein as these recent studies, and taking some important lessons from them, I use Word2Vec to estimate semantic change in key concepts associated with the UK’s constitution.^89^ This undertaking entails a number of choices about what textual data to use, how to divide the corpus for purposes of analysis and how to measure semantic change. These choices are explained below.

### B. The Corpus and Concepts of Interest

The texts of discourse chosen for this investigation are taken from Hansard’s records of parliamentary debate. There are good substantive reasons to focus on this material. Parliament is a salient and routine forum for discourse on constitutional affairs in the UK and, presumably, the notions of constitutional propriety that prevail there will be acutely influential on political practice. To paraphrase Griffith, Parliament is the most visible place where the UK’s constitution routinely ‘happens’.^90^ Parliamentary debate is also a concrete and pragmatic sort of discourse, largely directed to achieving the varied and variable political objectives of politicians: persuading or dissuading other parliamentarians or the public to support or oppose some policy or piece of legislation, reassuring constituents, defending allies or challenging opponents, and so on.^91^ For this reason, the discourse of parliamentary debate is bound to be responsive to changing circumstances. There are also compelling practical reasons to focus on debates in Parliament. Hansard publishes a complete and publicly accessible online textual record of these debates. Consequently, and in contrast to the discourse produced by Cabinet or Select Committee meetings, there is a massive repository of relevant textual data (spanning a time frame of over 200 years) which can be freely and remotely accessed, collated and indexed by the date of each debate.

To collect relevant text from Hansard, an automated ‘web scraping’ process was used.^92^ Rather than scraping the entirety of Hansard, which would have produced a gigantic but very computationally taxing corpus, the web scraping was targeted to return the text of all debates that make any reference to particular words or phrases of interest. These terms of interest included the obvious ones relating to the theoretical discussion above: ‘British constitution’, ‘unwritten constitution’, ‘parliamentary sovereignty’, ‘separation of powers, ‘the rule of law’, ‘devolution’ and ‘human rights’. To catch other debates of potential constitutional relevance, several additional terms were added to the web-scraping queries (eg ‘parliamentary democracy’, ‘civil liberties’, ‘judicial independence’, ‘judicial review’, ‘ministerial accountability’).^93^ Ultimately, these rounds of web scraping collected a massive (but still computationally manageable) corpus of texts: 11,160 parliamentary debates, comprising 200,625,472 words in total. It should be noted that this corpus includes debates from both the Lords and the Commons on both Public Bills and Private Member Bills, as well as non-legislative debates from both Houses. Because the inquiry at hand is concerned with parliamentary discourse in general, differences in the discourse across these various types of parliamentary debate are not explored here.

### C. Processing and Dividing the Corpus

To make the corpus amenable to word-embedding analysis, some preliminary text processing was required. All punctuation was stripped from the corpus and all letters were converted to a single case (lower case) for the algorithm to recognise all instances of a given word (regardless of case) as the same word.^94^ Furthermore, the text was processed so that phrases of interest would be assigned their own vectors. For example, because the algorithm needs to recognise that ‘the separation of powers’ is a single concept (and not four separate words), all instances of this phrase were converted to ‘the_separation_of_powers’. The same is true for ‘human rights’ (which was converted to ‘human_rights’).

In most cases, this sort of processing does not require much human intervention; there are ways to automatically detect commonly recurring phrases.^95^ In some cases, however, additional help from human judgment was required to prepare the text for analysis. This is true, for instance, in the case of ‘parliamentary sovereignty’. Not only does the algorithm need to recognise that this is a sign for a single concept (and not two independent words), but it should also treat other phrases that presumably express the same concept—‘the sovereignty of parliament’, ‘parliamentary supremacy’, etc—as effectively the same sign and therefore give them all a common vector (though it is possible that this assumption obscures some subtle semantic differences between these phrases). Consequently, the text was processed to collapse all such phrases into a single ‘word’ (‘parliamentary_sovereignty’). A similar intervention was required with respect to phrases that refer to the UK’s constitution. The word ‘constitution’ appears in Hansard hundreds of times in reference to the constitutions of other countries, especially Commonwealth countries (eg Canada, India, Jamaica). A vector for ‘constitution’ would therefore be influenced, in large part, by this generic sense of the concept. But the inquiry at hand is concerned with the concept of the UK’s constitution (and not ‘constitution’ in the generic sense). For this reason, I focused on commonly recurring phrases that unambiguously refer to the UK’s constitution, ie ‘British constitution’ and ‘our constitution’; these phrases were collapsed into a single term (‘british_constitution’) for the algorithm to assign them the same vector.^96^

Another important part of processing the corpus for analysis is deciding how to divide it into meaningful slices of time. The corpus needs to be divided in a way that helps to answer the research questions at hand; the temporal cut points should track expectations relating to the concepts of interest. But there is also a balance to be struck between dividing the corpus into the smallest possible sub-corpora, to maximise granular information on semantic change, and giving the Word2Vec algorithm sufficient text to produce robust word embeddings. In light of these concerns, I divided the corpus of debates according to turnover in control of government. Thus, debates from the years of Conservative government from 1979 to 1997 are grouped into one sub-corpus (‘The Thatcher-Major Era’) and debates from New Labour’s period in government from 1997 to 2010 are grouped into another (‘The New Labour Era’). Thereafter, the corpus is divided into the period of Conservative–Liberal Democrat coalition government from 2010 to 2015 (‘The Coalition Era’) and the period of Conservative government from 2015 up to the election in November 2019 (which, for lack of a better name, I call ‘The Post-Coalition Era’). Dividing the corpus in this way tracks distinct agendas of constitutional reform^97^ and yields four sub-corpora that are large enough for machine learning: the Thatcher-Major Era sub-corpus is 40,615,581 words long; the New Labour Era sub-corpus comprises 46,242,503 words; the Coalition Era sub-corpus comprises 22,082,712 words; and the Post-Coalition Era sub-corpus comprises 22,972,042 words.

### D. Measuring Change in Constitutional Resonance

The present inquiry requires a measure of what I have called constitutional resonance—that is, the degree to which a concept is semantically related to the UK’s constitution. To this end, constitutional resonance is treated as a latent variable which, although not directly observable, can be estimated by examining how people (in this case, parliamentarians) speak about the concepts of interest. The more that talking about a concept is akin to talking about the constitution (and vice versa), the closer that concept’s vector will be embedded to the vector representing the UK’s constitution. The cosine similarity score for the associated vectors is therefore an estimate of a concept’s constitutional resonance.

I employed two additional techniques to enhance the robustness of these estimates. First, multiple models (25) were created for each sub-corpus so that averages (ie arithmetic means) of vector cosine similarity scores for each sub-corpus can be reported alongside estimates of uncertainty.^98^ This extra modelling is time consuming (each model takes several hours to train), but the machine learning process has some inherent random variability built into it that, unless accounted for in this way, may threaten the replicability of the inferences that we hope to elicit from the models.^99^ Creating many models and averaging cosine similarity scores across them yields estimates that are more robust to this variability.^100^ Second, each model was trained through a technique called ‘bootstrap resampling’, causing the models to be trained on random samples of sentences from each sub-corpus (as opposed to the entirety of sentences from each sub-corpus as they originally appear in Hansard).^101^ The idea here is to mitigate the undue influence that incidental variation in the size and/or composition of sub-corpora might otherwise have on the results. The sub-corpus for the New Labour Era, for example, is much larger (46,242,503 words) than that for the Coalition Era (22,082,712 words). Consequently, a single (and perhaps idiosyncratic) debate from the Coalition Era might have an outsized influence relative to a single (and perhaps more representative) text from the New Labour Era. Moreover, the frequency with which the concepts of interest occur in parliamentary debate varies over time and so a concept might appear to have gained (or lost) constitutional resonance simply because it has become more (or less) prevalent in the relevant debate texts. Bootstrap resampling compensates for these issues. By averaging vector cosine similarity scores across multiple samples, the resultant estimates of semantic change will be robust to incidental differences in the size and/or composition of the sub-corpora.^102^ Thus, for example, if the models suggest that a concept such as ‘human rights’ became more constitutionally resonant during the New Labour period, this would not be because of any single debate text or because that concept became more prevalent in debate. Rather, the estimated change in constitutional resonance will reflect a change in the *typical* discourse surrounding that concept.

The techniques explained above enhance the robustness of the estimates for each sub-corpus, but they do not address the problem of how to make those results comparable across time. Comparability of diachronic word-embedding models can be problematic: if each sub-corpus is treated as a separate unrelated textual universe, then the resultant models will lack a common vector space as a frame of reference for comparison.^103^ To get around this problem, I employed a chronological training method recently pioneered and empirically validated by Emma Rodman.^104^ This method begins with a foundational model trained on the full corpus of text to fix a stable frame of reference, followed by a series of models that are retrained for each of the sub-corpora in chronological order.^105^ Comparing the performance of this approach with the available alternatives in empirical trials, Rodman finds that this method of chronological training is superior: the cumulative information in each chronological training cycle ‘appears to allow the model to reproduce the overall structure of semantic shifts in the corpus with higher fidelity’.^106^ I performed an independent validation of Rodman’s chronological training method, in addition to several more general validity tests.^107^

## 4. Charting Semantic Change in Constitutional Concepts

To estimate a concept’s constitutional resonance, I compute the cosine similarity between that concept’s vector and the vector for ‘british_constitution’. The expectation here is that these vectors will become more similar to one another as the concept in question becomes more constitutionally resonant. In other words, as a concept’s constitutional resonance increases, that concept’s vector will become more proximate to the vector for ‘british_constitution’ within the semantic map of the word-embedding model. It is important to recall here that word-vector similarity is not based simply on the frequency with which two terms appear within the same sentences. Rather, it reflects similarities (and differences) in the language that accompanies those terms. More specifically, in this instance, cosine similarity will be indicative of the degree to which talking about each of the concepts of interest is (or is not) akin to talking about the UK’s constitution. This measure allows for a continuum of constitutional resonance (from ‘–1’ to ‘1’). A cosine similarity score of ‘–1’ would imply that the meaning of a concept is utterly inapposite to the meaning of the constitution. Conversely, a cosine similarity score of ‘1’ would imply that the concept is perfectly synonymous with the UK’s constitution, ie that the two are so semantically similar there is no appreciable difference in how they are spoken about. For example, to the extent that the UK’s constitution has often been equated with its primarily ‘unwritten’ form, one might expect the language that tends to accompany ‘unwritten_constitution’ to be very similar to the language that tends to accompany ‘british_constitution’. The cosine similarity score of the associated word vectors should therefore indicate a relatively high degree of constitutional resonance.

**Figure 1:**
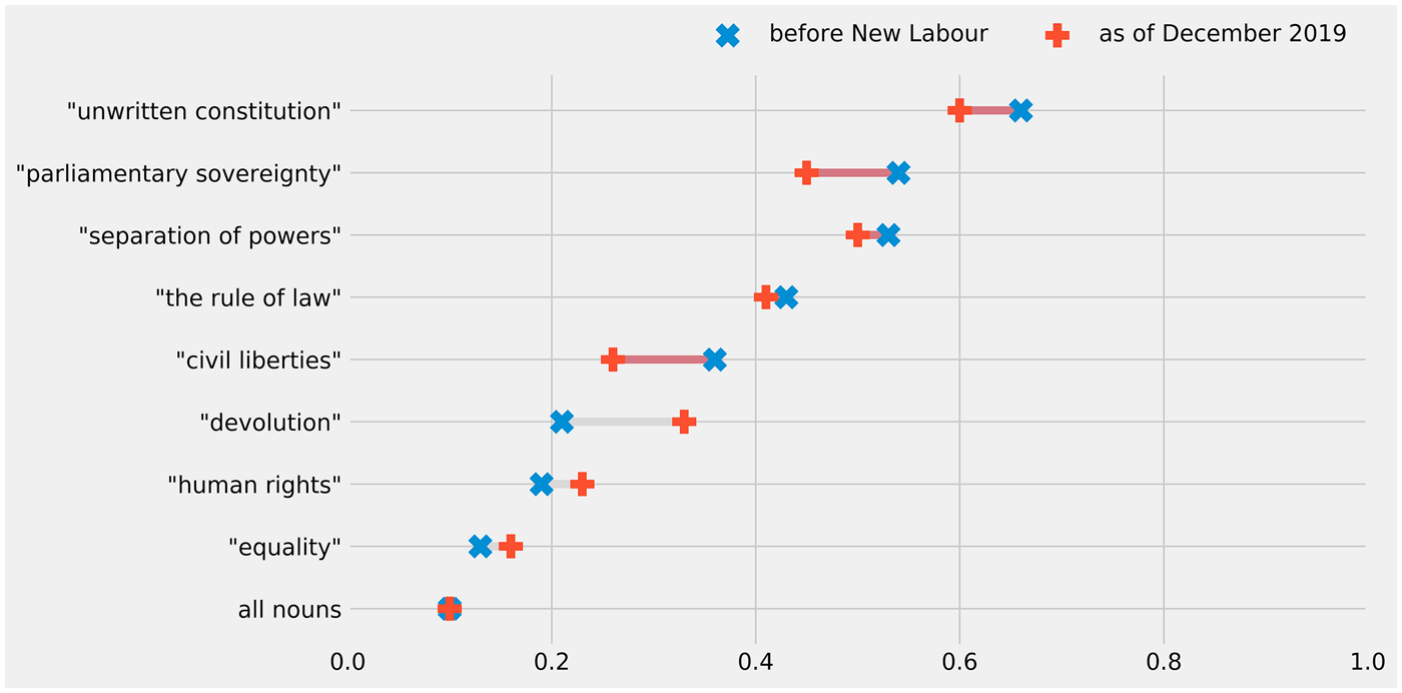
Long-term change in constitutional resonance of concepts (as measured by vector cosine similarity to ‘british_constitution’).

Figure 1 charts the estimated constitutional resonance for each concept of interest. The average vector cosine similarity to ‘british_constitution’ for all nouns appearing in the corpus is included to provide a ‘baseline’ for comparison. For purposes of further comparison, ‘equality’ and ‘civil_liberties’ are also included in this plot.

The first thing to note about these results is that the average constitutional resonance for all nouns shows precisely what one would expect, ie the lowest cosine similarity scores to ‘british_constitution’ across time, and these estimates are steady (at about 0.1 cosine similarity). All the other concepts being examined are estimated to be more constitutionally resonant than this baseline. Interestingly, one can also see that ‘equality’ is estimated to have a consistently low constitutional resonance relative to the other terms under consideration; its cosine similarity with ‘british_constitution’ is only ever slightly greater than the average score for all nouns (although ‘equality’ does appear to have gained a small but statistically significant degree of constitutional resonance since 1997).^108^

**Figure 2:**
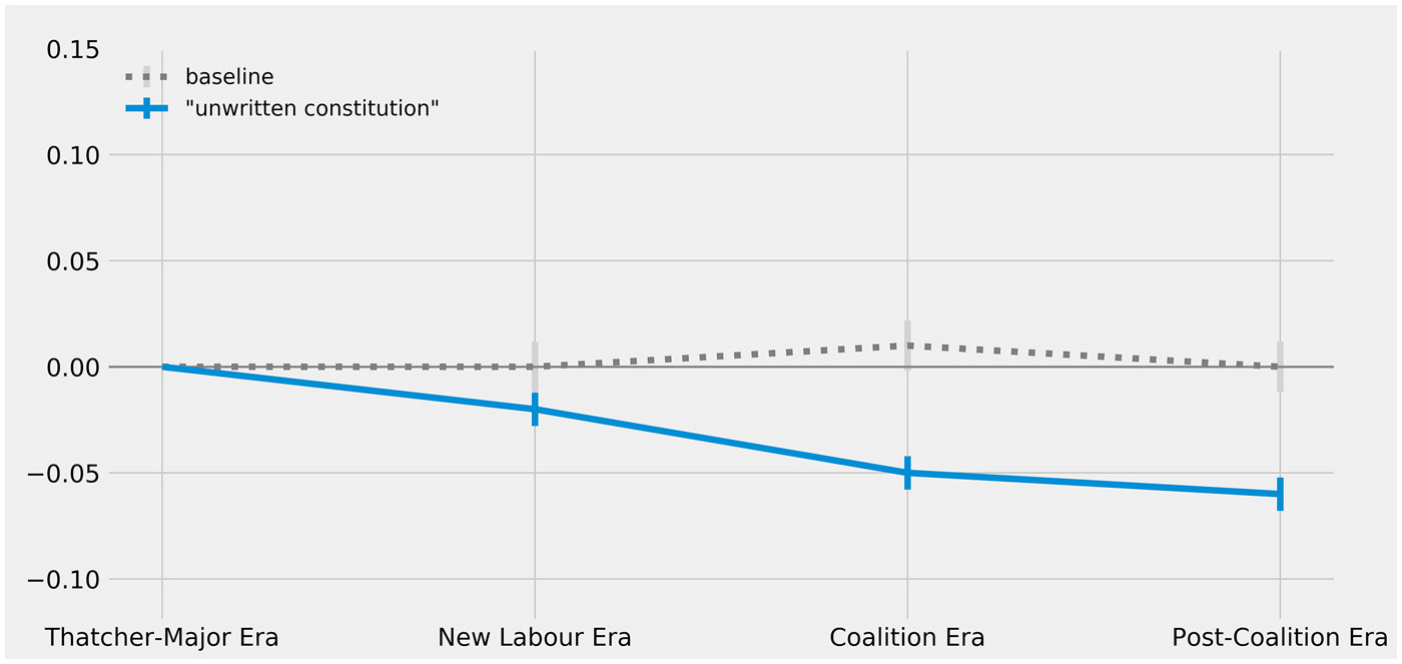
Change over time in constitutional resonance of ‘unwritten_constitution’ (as measured by change in vector cosine similarity to ‘british_constitution’).

The next thing to note is that ‘unwritten_constitution’ is consistently estimated to be the most semantically similar concept to ‘british_constitution’. As was noted earlier, this is broadly what one would expect to see (given that the UK’s constitution has often been equated with a primarily unwritten form). It should be highlighted, however, that the models also suggest a steady and significant decline over time in the constitutional resonance of ‘unwritten_constitution’. Figure 2 plots these estimated changes (with error bars for their respective 95% confidence intervals), alongside a baseline expectation based on the average change in constitutional resonance for all nouns.

As Figure 2 illustrates, there is a statistically significant decline in estimated cosine similarity for ‘unwritten_constitution’ and ‘british_constitution’ during the New Labour Era, suggesting that the UK’s constitution became less equated with the concept of an ‘unwritten constitution’ during this time. This diminished constitutional resonance appears to be sustained over the course of the next two eras; it is estimated to drop again (to a statistically significant degree) with the Coalition Era and again (though not to a statistically significant degree) during the Post-Coalition Era. These results are broadly consistent with scholarly intuitions canvassed earlier about the general decline of the informal and primarily unwritten sense of the UK’s constitution in the wake of New Labour’s reforms, though they do not yet provide confirmation of more specific hypotheses about changes in the constitutional resonance of particular concepts.

**Figure 3:**
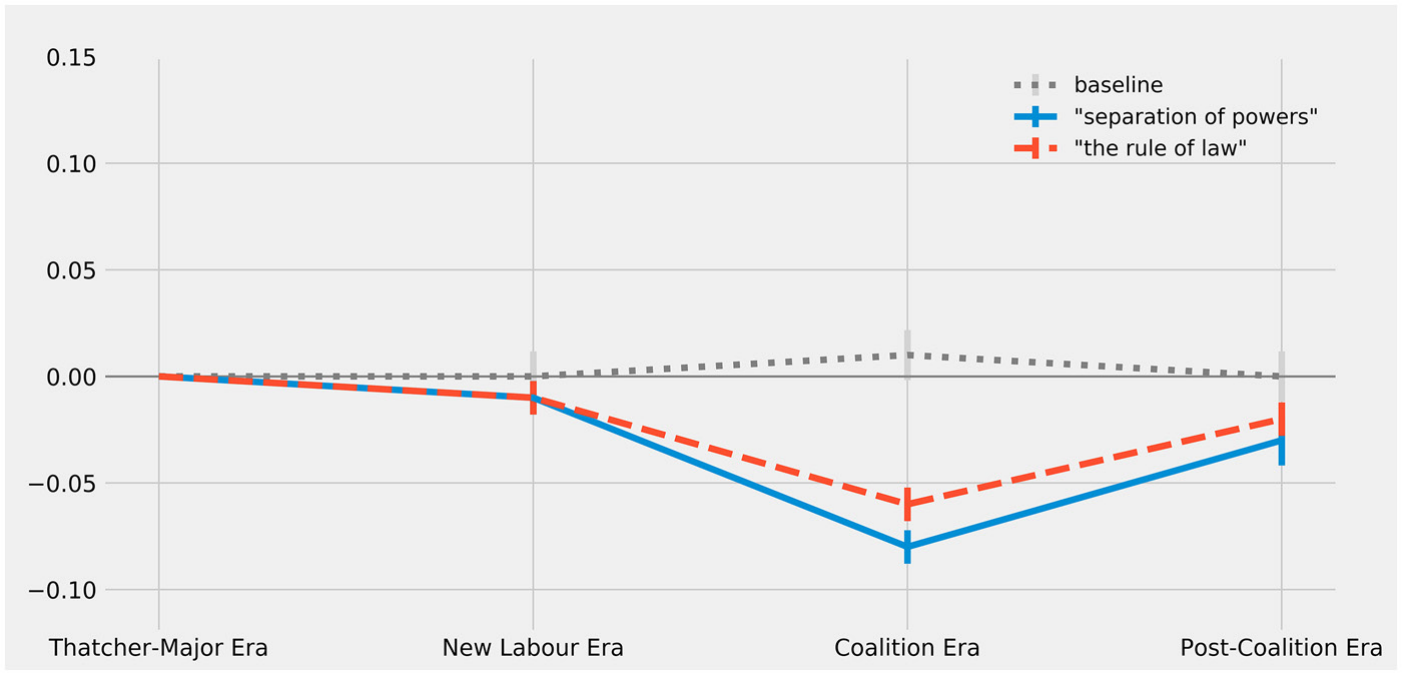
Change over time in constitutional resonance of ‘separation_of_powers’ and ‘the_rule_of_law’ (as measured by change in vector cosine similarity to ‘british_constitution’).

Turning next to these more specific hypotheses, some of them are plainly *not* supported by the results of the word-embedding models. Contrary to theoretical expectations, neither ‘the_rule_of_law’ nor ‘separation_of_powers’ appears to have gained any constitutional resonance since New Labour’s reforms. If anything, the models suggest that these two concepts probably lost a degree of constitutional resonance during the Coalition Era. Figure 3 plots the results alongside the baseline average change in constitutional resonance for all nouns.

Although the models strongly suggest that New Labour’s reforms did *not* amplify the constitutional resonance of the concepts of the rule of law and the separation of powers, it is not clear what else should be inferred from the estimates. On the one hand, the models suggest that the discourse surrounding these concepts palpably diverged from discourse about the constitution, at least during the Coalition Era. On the other hand, both concepts seem to have regained most of their original constitutional resonance during the Post-Coalition Era, which suggests that the apparent decline during the preceding era may have been an ephemeral anomaly. In the absence of any theoretical expectation for these fluctuating estimates, the most that one can responsibly conclude here is that there has been a modest net decline in the constitutional resonance of the concepts of the rule of law and the separation of powers. Furthermore, in relative terms, this net decline is too small to signal much of a disruption to the overall conceptual scheme of the UK’s constitution; in comparison with the other concepts under consideration, the concepts of the rule of law and separation of powers appear to have enjoyed relatively high degrees of constitutional resonance before New Labour’s reforms and they have enjoyed comparable (if slightly diminished) degrees of constitutional resonance during the most recent period under consideration.

**Figure 4:**
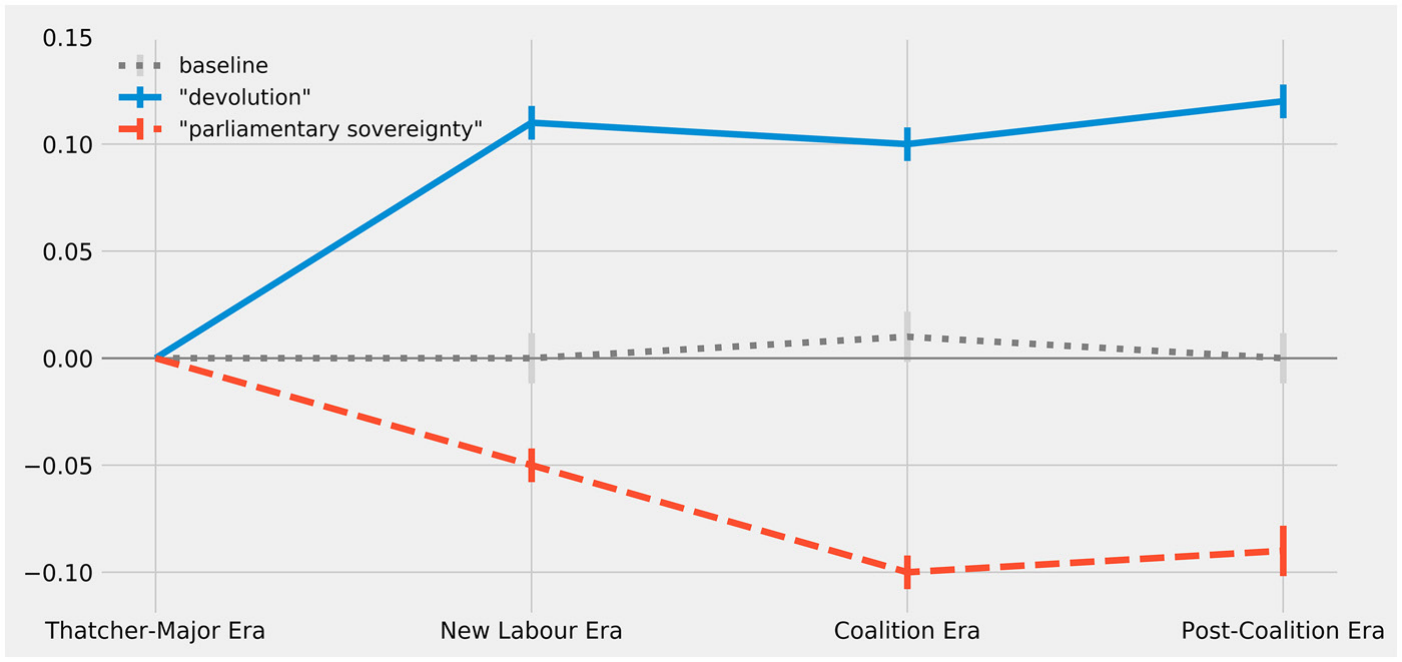
Change over time in constitutional resonance of ‘parliamentary_sovereignty’ and ‘devolution’ (as measured by change in vector cosine similarity to ‘british_constitution’).

Other hypotheses about semantic change are vindicated by the results of the word-embedding models. Figure 4 plots the estimated changes in constitutional resonance for ‘parliamentary_sovereignty’ and ‘devolution’ alongside the baseline average change for all nouns.

As hypothesised, ‘devolution’ is estimated to have gained a higher degree of constitutional resonance during the New Labour Era, and this gain is relatively large and statistically significant. This substantial increase in constitutional resonance should not be surprising (indeed, one would rightly question the validity of the models if ‘devolution’ was not estimated to have gained some constitutional resonance over this period). Apparently, following the creation of the devolved legislatures, the way parliamentarians speak about ‘devolution’ became more akin to discourse about the UK’s constitution. The results for ‘parliamentary_sovereignty’ are even more striking because they concern a more speculative hypothesis. As can be seen in Figure 4, the estimated constitutional resonance of ‘parliamentary_sovereignty’ significantly drops during the New Labour Era and again during the Coalition Era, and this lower degree of constitutional resonance persists into the Post-Coalition Era. These results would seem to confirm the expectation that the concept of parliamentary sovereignty has a now-diminished constitutional significance; as was hypothesised, the discourse has changed such that the concept of parliamentary sovereignty now appears to be less associated with the meaning of the constitution than it was prior to New Labour’s reforms. Indeed, as Figure 1 foreshadowed, this decline has been so pronounced that the relative hierarchy of constitutional resonance, as between the concepts of parliamentary sovereignty and the separation of powers, appears to have been reversed.

**Figure 5:**
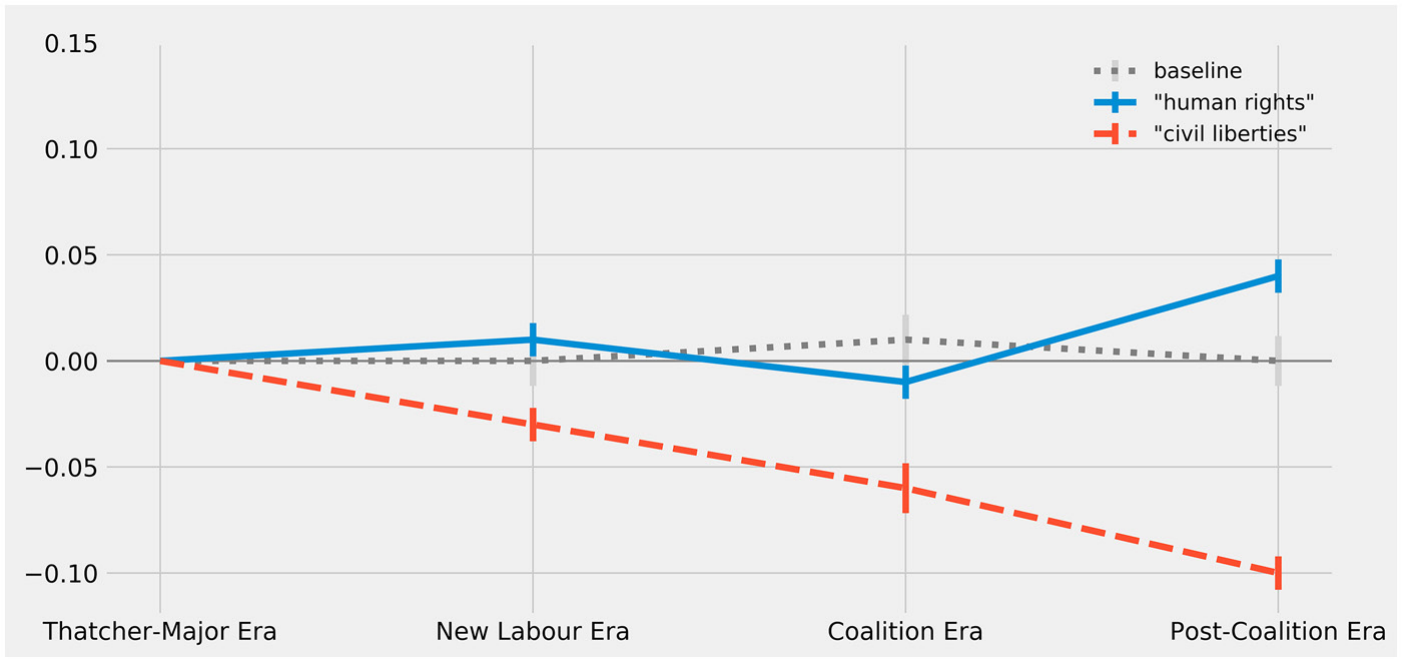
Change over time in constitutional resonance of ‘human_rights’ and ‘civil_liberties’ (as measured by change in vector cosine similarity to ‘british_constitution’).

The results with respect to ‘human_rights’ are less conclusive. Figure 5 plots the estimated changes in constitutional resonance for ‘human_rights’ and ‘civil_liberties’ alongside the baseline average change in constitutional resonance for all nouns.

Given the fluctuating estimates, one cannot draw a firm conclusion about any enduring trend relating to the concept of human rights. Consistent with what was hypothesised, the concept appears to have acquired a greater degree of constitutional resonance after the New Labour period, but this increase comes later than expected. This delayed gain in constitutional resonance is puzzling: why would human rights have taken on a greater constitutional resonance during a period in which the Conservative government was proposing to repeal the Human Rights Act?

One plausible answer to this puzzle is that it is only *after* David Cameron’s government made concrete proposals for the repeal of the Human Rights Act (following the Conservative victory in the 2015 election) that some parliamentarians—in defence of the Act—really began to speak about human rights as ‘part of the constitutional architecture of the United Kingdom’.^109^ Hence, parliamentary speech during this period (2015–19) includes claims that it would be unconstitutional for Parliament to unilaterally repeal the Human Rights Act 1998. One strand of this defence relates to devolution. For example, Joanna Cherry MP argued in the House of Commons that the topic of human rights—because it is not an explicitly reserved matter for Westminster—must be understood as a devolved area of competence and, consequently, that the repeal of the Human Rights Act would require the consent of the Scottish Parliament.^110^ Another strand of this defence relates to the Good Friday Agreement; parliamentary speech during the Post-Coalition Era includes claims that the repeal of the Human Rights Act would breach the terms of the constitutional settlement for Northern Ireland and the associated international treaty with the Republic of Ireland.^111^ It would seem, then, that the very act of opposing proposals for the repeal of the Human Rights Act may have brought the constitutional significance of human rights to the foreground of the discourse. It is still too early to tell if the recently amplified constitutional resonance of human rights detected here is a signal of an enduring change. It should be noted, however, that the estimates also show a consistent and statistically significant decline in the constitutional resonance of ‘civil_liberties’ over the last few decades (see Figure 5), a sign perhaps that the more modern concept of human rights may ultimately supplant the more traditional concept of civil liberties in the conceptual scheme of the UK’s constitution.

## 5. Concluding Discussion

Walter Bagehot once observed that one of the great difficulties in writing about the UK’s constitution is that it is a ‘living’ object and thus subject to ‘constant change’.^112^ This difficulty is even greater for contemporary scholars because the UK’s constitution is more complex and less settled now than it was in Bagehot’s day; the constitution comprises more moving parts, several of which have either been reformed recently and/or may be subject to reform in the future. It is a daunting task to canvass the myriad formal changes that have taken place and perhaps an even greater challenge to take stock of the informal changes that accompany shifting understandings.

To meet this challenge, this article has leveraged recent advances in machine learning to analyse parliamentary discourse and measure semantic change in several concepts associated with the constitution. The results support some bold claims about informal constitutional change in the UK. The more controversial of these relate to the nature of the constitution and the significance of the concept of parliamentary sovereignty: the extent to which the UK’s constitution is equated with an ‘unwritten’ form has apparently waned over time, while the constitutional resonance of parliamentary sovereignty appears to have also declined significantly. In short, the meaning of the UK’s constitution seems to have changed in ways that broadly track theoretical expectations about the consequences of New Labour’s reforms, at least in so far as this meaning might be manifested in parliamentary debate. But the findings also suggest that some claims about the ‘new’ constitution may be overstated. Claims that the concept of parliamentary sovereignty has been ‘permanently undermined’ or that the constitution of Bagehot and Dicey ‘no longer exists’^113^ are belied by apparent semantic continuity in the conceptual scheme of the constitution. Although the constitutional resonance of parliamentary sovereignty may have been diminished, that concept still appears to be very closely connected to the meaning of the constitution, relative to other concepts, within parliamentary discourse; for instance, the evidence presented here suggests that parliamentary sovereignty is still a more constitutionally resonant concept than the concepts of the rule of law and human rights.

It must be stressed that the findings presented here specifically concern *parliamentary* discourse. Presumably, semantic changes in this discourse are especially consequential; in so far as these changes shape or reflect parliamentarians’ subjective beliefs about constitutional propriety, they may influence what is done (or not done) by Parliament. But these beliefs are not sacrosanct, and contestation about them can migrate from Parliament to other fora—the Supreme Court’s recent decision in *Cherry/Miller (No 2)* on prorogation is a recent and vivid illustration of this fact.^114^ Indeed, it should be emphasised that Parliament is not the only forum for discourse about the constitution, and one should not assume that semantic changes in parliamentary discourse will necessarily be mirrored in the discourses of the judiciary or the legal academy (though they might be). A similar analysis of the discourses of these other communities may yield different results because these communities are differently involved with the constitution. These other communities are also subject to different norms constraining what can and cannot be said. A retired judge writing in an extrajudicial capacity, for instance, is probably at liberty to say things about parliamentary sovereignty that an active judge cannot readily write into her next decision. Legal scholars can say very speculative things about the constitution, but they are nonetheless subject to the discipline of peer review.^115^ Relative to these other discourse communities, parliamentarians may be much less constrained in how they speak about the constitution. Furthermore, the foregoing analysis has not investigated differences in constitutional resonance across types of parliamentary debate. It may be, for example, that some concepts are more constitutionally resonant in the context of legislative debates than they are in the context of non-legislative debates.

These limitations aside, the importance of the analysis presented here is not exhausted by the extent to which the results support (or disappoint) one theory of constitutional change or another. The analysis also demonstrates a novel way in which the study of the constitution can be extended beyond formal institutional questions. As an ideational construction, the UK’s constitution is constantly being reproduced and reimagined through the discursive practices of parliamentary debate, legal argument, judicial reasoning, university teaching and scholarship in journals like this one. All of these practices matter and should be studied, even if some of them are more immediately consequential than others. An array of different research methods, including newer computational methods like those used here, can help us learn about the UK’s ever-changing constitution, especially in so far as subtler conceptual aspects of constitutional change are of interest.

## Figures and Tables

**Figure 6:**
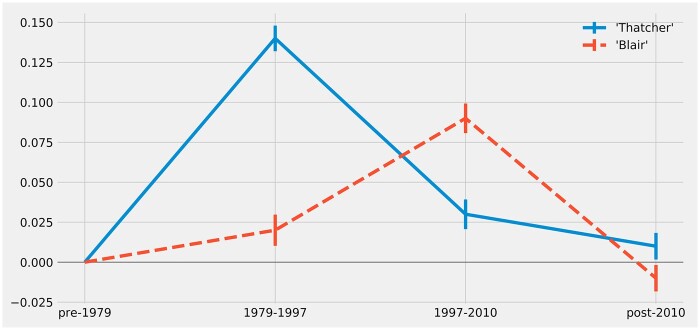
Change over time in semantic relatedness of ‘Prime Minister’ to ‘Thatcher’ and ‘Blair’ (as measured by change in vector cosine similarity).

## Footnotes

1 See generally Vernon Bogdanor, *The New British Constitution* (Bloomsbury Publishing 2009); Robert Stevens, *The English Judges: Their Role in the Changing Constitution* (Bloomsbury Publishing 2002); Graham Gee and others, *The Politics of Judicial Independence in the UK’s Changing Constitution* (CUP 2015); Jeffrey Jowell and Colm O’Cinneide (eds), *The Changing Constitution* (9th edn, OUP 2019).

2 See Northern Ireland Act 1998; Scotland Act 1998; and Government of Wales Act 1998.

3 Human Rights Act 1998.

4 House of Lords Act 1999.

5 Constitutional Reform Act 2005, pts 2, 4 and 5.

6 Constitutional Reform Act 2005, pt 3.

7 Bogdanor (n 1).

8 Nicholas Bamforth, ‘Current Issues in United Kingdom Constitutionalism: An Introduction’ (2011) 9 ICON 79.

9 Neil Walker, ‘Our Constitutional Unsettlement’ (2014) PL 529.

10 See Labour Party, *The Governance of Britain* (London 2007); Labour Party, *The Coalition: Our Programme for Government* (London 2010); Conservative and Unionist Party, *Get Brexit Done: Unleash Britain’s Potential* (London 2019).

11 The European Union (Withdrawal) Act 2018. Arguably, the second most consequential constitutional reform of the post-Blair era was the Fixed Term Parliament Act 2011.

12 Zachary Elkins, Tom Ginsburg and James Melton, *The Endurance of National Constitutions* (CUP 2009).

13 For the inception of the theory of constitutional statutes, see *Thoburn v Sunderland City Council* [2002] EWHC 195 (Admin), [2003] QB 151. For the subsequent endorsement of this theory at the UK Supreme Court, see *H v Lord Advocate* [2013] 1 AC 413. For academic commentary, see David Feldman, ‘The Nature and Significance of “Constitutional” Legislation’ (2013) 129 LQR 343; Farrah Ahmed and Adam Perry, ‘Constitutional Statutes’ (2017) 37 OJLS 461.

14 Jeffrey Jowell and Dawn Oliver, ‘Editor’s Introduction’ in Jeffrey Jowell and Dawn Oliver (eds), *The Changing Constitution* (7th edn, OUP 2011) 3. There is a large academic literature on the political constitution and political constitutionalism, starting with JAG Griffith, ‘The Political Constitution’ (1979) 42 MLR 1. For further discussion, see Adam Tomkins, ‘In Defence of the Political Constitution’ (2002) 22 OJLS 157; Thomas Poole, ‘Tilting at Windmills? Truth and Illusion in “The Political Constitution”’ (2007) 70 MLR 250; Graham Gee and Grégoire CN Webber, ‘What Is a Political Constitution?’ (2010) 30 OJLS 273.

15 Sidney Low, *The Governance of England* (T Fisher Unwin 1904) 19.

16 Mark Elliott, ‘Judicial Power and the United Kingdom’s Changing Constitution’ (2017) 36 UQLJ 273.

17 As Mark Elliot puts it, a change in the practice raises the question of ‘a challenge to or a shift in the prevailing consensus’. ibid 274.

18 Bogdanor makes a similar point: ‘It adds to the difficulties facing the constitutional lawyer that he or she must capture something that is in the process of change even as he or she writes about it.’ Vernon Bogdanor, ‘Constitutional Law and Politics’ (1987) 7 OJLS 454, 457.

19 Other methods that are broadly ‘computational’ in nature have been used in British public law scholarship. See eg the use of a computational ‘dictionary-based’ method in Michael Blackwell, ‘Indeterminacy, Disagreement and the Human Rights Act: An Empirical Study of Litigation in the UK House of Lords and Supreme Court 1997–2017’ (2020) 83 MLR 285; and the use of ‘Bayesian ideal point estimation’ in TT Arvind and Lindsay Stirton, ‘Legal Ideology, Legal Doctrine and the UK’s Top Judges’ (2016) PL 418. For examples of legal scholarship from outside the UK that use methods similar to the word-embedding method used in this article, see Elliott Ash and Daniel L Chen, ‘Case Vectors: Spatial Representations of the Law Using Document Embeddings’ in Michael Livermore and Daniel Rockmore (eds), *Law as Data* (Santa Fe Institute Press 2019); David S Law, ‘Constitutional Archetypes’ (2016) 95 Tex L Rev 153; Douglas Rice, Jesse H Rhodes and Tatishe Nteta, ‘Racial Bias in Legal Language’ (2019) 6 Research and Politics 1.

20 Albert Venn Dicey, *Introduction to the Law of the Constitution* (8th edn, Macmillan 1915) 3.

21 For discussion and critique of this lingering influence, see Martin Loughlin and Stephen Tierney, ‘The Shibboleth of Sovereignty’ (2018) 81 MLR 989. See also Nicholas Barber, ‘The Afterlife of Parliamentary Sovereignty’ (2011) 9 ICON 144.

22 For an overview of the challenges to parliamentary arising out of devolution, the Human Rights Act 1998, common law constitutionalism and the UK’s past membership in the EU, see Michael Gordon, *Parliamentary Sovereignty in the UK Constitution: Process, Politics and Democracy* (Hart Publishing 2015).

23 Bogdanor (n 1).

24 ibid 271.

25
*R (Factortame Ltd) v Secretary of State for Transport (No 2)* [1991] 1 AC 603.

26 Bogdanor (n 1) 284.

27 ibid 261, 276.

28 ibid 89.

29 ibid 62.

30 ibid 2.

31 See eg Mark Bevir, ‘The Westminster Model, Governance and Judicial Reform’ (2008) 61 Parliamentary Affairs 559; Roger Masterman ‘Labour’s “Juridification” of the Constitution’ (2009) 62 Parliamentary Affairs 476. See also Erin Delaney, ‘Judiciary Rising: Constitutional Change in the United Kingdom’ (2013) 108 Northwestern University Law Review 543.

32 See the now-famous obiter dicta in *R (Jackson) v Attorney General* [2005] UKHL 56, [102] (Lord Steyn), [159] (Baroness Hale) and [104] (Lord Hope). Lord Steyn’s comments in *Jackson* are especially striking—he proposed that the UK’s constitution was no longer ‘uncontrolled’, he singled out the Human Rights Act in particular for having inaugurated what he called a ‘new legal order’ and he speculated that ‘it is not unthinkable that circumstances could arise where the courts may have to qualify a principle established on a different hypothesis of constitutionalism’. More recent cases have echoed (or at least intimated support for) some of the ideas expressed in *Jackson*. See *AXA General Insurance Ltd v HM Advocate* [2011] UKSC 46, [50] (per Lord Hope); *R (Cart) v Upper Tribunal* [2011] UKSC 28, [73] (per Lord Phillips). For discussion, see Tom Mullen, ‘Reflections on Jackson v Attorney General: Questioning Sovereignty’ (2007) 27 LS 1.

33 Aileen McHarg, ‘Reforming the United Kingdom Constitution: Law, Convention, Soft Law’ (2008) 71 MLR 853.

34 Alison Young, *Parliamentary Sovereignty and the Human Rights Act* (Bloomsbury Publishing 2008). Cf Aileen Kavanagh, *Constitutional Review under the UK Human Rights Act* (CUP 2009).

35 Richard Bellamy, ‘Political Constitutionalism and the Human Rights Act’ (2011) 9 ICON 86.

36 Neil Walker, ‘Our Constitutional Unsettlement’ (2014) 7 PL 529.

37
*Jackson* (n 32) [102] (Lord Steyn).

38 For an explanation of the idea of an ‘essentially contested’ concept, looking at the rule of law specifically, see Jeremy Waldron, ‘Is the Rule of Law an Essentially Contested Concept (In Florida)?’ (2002) Law and Philosophy 137.

39 Some readers will be reminded of Wittgenstein’s comments on meaning and use in Ludwig Wittgenstein, *Philosophical Investigations* (John Wiley & Sons 2009). Skinner’s approach to the history of ideas shares a similar theory of meaning, apparently inspired by Wittgenstein. See Quentin Skinner, ‘Meaning and Understanding in the History of Ideas’ (1969) 8 History and Theory 3. This sort of historicisation of concepts is prefigured in the philosophy of Nietzsche. See Friedrich Nietzsche, *On the Genealogy of Morals* (Horace Samuel tr, Dover Publications 2003) 53.

40 Strictly speaking, as Quentin Skinner points out, changes of this kind are not really changes to the concepts themselves; they are perhaps more accurately described as ‘transformations in the applications of the terms by which our concepts are expressed’. Quentin Skinner, *Visions of Politics*, vol 1 (CUP 2002) 179.

41 Gemma Boleda, ‘Distributional Semantics and Linguistic Theory’ (2020) 6 Annual Review of Linguistics 213.

42 ibid.

43 See Matthias Urban, ‘Lexical Semantic Change and Semantic Reconstruction’ in Claire Bowern and Bethwyn Evans (eds), *The Routledge Handbook of Historical Linguistics* (Routledge 2015).

44 A convergence in denotation may arise because of a perceived similarity in the original referents of two words (ie *metaphor*) or because of a contiguity of their referents in space and time (ie *metonym*). See ibid 374–5.

45 Helen Saberi, *Tea: A Global History* (Reaktion Books 2010) 106.

46 The terms ‘semantic relatedness’ and ‘semantic similarity’ are often used interchangeably, but some computational linguists treat ‘semantic similarity’ as a special type of ‘semantic relatedness’, approximating a relation of synonymity. See the discussion in Sebastien Harispe and others, *Semantic Similarity from Natural Language and Ontology Analysis* (Morgan & Claypool 2015) 10.

47 Martin Loughlin, ‘In Search of the Constitution’ (9 December 2019) LSE Legal Studies Working Paper 19/2019 <https://ssrn.com/abstract=3501014> accessed 24 May 2021.

48 ibid 20.

49 Martin Loughlin, *The British Constitution: A Very Short Introduction* (OUP 2013) 248.

50 ibid 245–7. See also Thomas Poole, ‘Back to the Future? Unearthing the Theory of Common Law Constitutionalism’ (2003) 23 OJLS 435.

51 See eg Mark Elliott, ‘Parliamentary Sovereignty and the New Constitutional Order: Legislative Freedom, Political Reality and Convention’ (2006) 22 LS 340. See also Jowell and Oliver (n 14).

52 Mark Elliot, ‘Parliamentary Sovereignty in a Changing Constitutional Landscape’ in Jowell and O’Cinneide (n 1) 30.

53 Dicey (n 20) 3.

54 Bogdanor (n 1) 282.

55 Loughlin and Tierney (n 21).

56 See Kate Malleson, ‘The Rehabilitation of Separation of Powers in the United Kingdom’ in Leny E de Groot van Leeuwen and Wannes Rombouts (eds), *Separation of Powers in Theory and Practice* (Wolf Legal Publishers 2010).

57 See Gee and others (n 1).

58 Roger Masterman and Se-shauna Wheatle, ‘Unpacking Separation of Powers: Judicial Independence, Sovereignty and Conceptual Flexibility in the UK Constitution’ (2017) PL 469.

59 Constitutional Reform Act 2005, s 1.

60
*R (on the application of Privacy International) (Appellant) v Investigatory Powers Tribunal and others (Respondents)* [2019] UKSC 22; see also *R (UNISON) v Lord Chancellor* [2017] UKSC 51; *AXA General Insurance Limited and others (Appellants) v The Lord Advocate and others (Respondents) (Scotland)* [2011] UKSC 46.

61 Jeffrey Jowell, ‘The Rule of Law’ in Jowell and O’Cinneide (n 1) 9.

62 ibid. For an influential and relatively ‘thick’ account of the rule of law, see Tom Bingham, *The Rule of Law* (Penguin 2011).

63 On the history of proposals for and debate about devolution for Scotland, see Aileen McHarg, ‘Devolution in Scotland’ in Jowell and O’Cinneide (n 1) 273–9.

64 See eg Richard Leonard, ‘Towards Home Rule for Scotland’ *Tribune* (18 February 2020), <https://tribunemag.co.uk/2020/02/home-rule-for-scotland> accessed 24 May 2021.

65 See Hansard’s graphs of references over time, at ‘“Devolution” References, 01/01/1800–02/08/2020’ (UK Parliament) <https://hansard.parliament.uk/search?startDate=1800-01-01&endDate=2020-08-02&searchTerm=%22devolution%22&partial=False> accessed 24 May 2021 and ‘“Home rule” References, 01/01/1800–02/08/2020’ (UK Parliament) <https://hansard.parliament.uk/search?startDate=1800-01-01&endDate=2020-08-02&searchTerm=%22home%20rule%22&partial=False> accessed 24 May 2021.

66 Northern Ireland Act 1998, s 79 and Sch 10; Scotland Act 1998, s 98 and Sch 6; Government of Wales Act 2006, Sch 9.

67 Scotland Act 2016, s 2.

68 Wales Act 2017, s 2.

69
*R (on the application of Miller and another) v Secretary of State for Exiting the European Union* [2017] UKSC 5.

70 See Scotland Act 2016, s 1; Wales Act 2017, s 1. The status of the Northern Ireland Assembly is different because its continued existence is tied to the Good Friday Agreement of 1998 and therefore underpinned by an international treaty between the UK and Ireland. See The Belfast Agreement 1998, ‘Strand One’ and Annex, ‘Agreement between the Government of the United Kingdom of Great Britain and Northern Ireland and the Government of Ireland’.

71 On the evolution of the ‘civil liberties’ discourse, see generally Conor Gearty, *Civil Liberties* (OUP 2007).

72 See generally Colm O’Cinneide, ‘Human Rights and the UK Constitution’ in Jowell and O’Cinneide (n 1). For an argument about the relatively recent origins of the concept of human rights, see generally Samuel Moyn, *The Last Utopia: Human Rights in History* (Harvard UP 2012).

73 Human Rights Act 1998, ss 3 and 6.

74 See Human Rights Act 1998, s 6(1). For commentary on this aspect of the Human Rights Act, see O’Cinneide (n 72) 78.

75 ibid, s 19.

76 <www.parliament.uk/business/committees/Committees-A-Z/joint-select/human-rights-committee/> accessed 24 May 2021.

77 HL Deb 18 December 2002, vol 642, col 685.

78 See David M Berry and Anders Fagerjord, *Digital Humanities: Knowledge and Critique in a Digital Age* (Wiley 2017).

79 For general overviews of the use of computational methods in legal scholarship, see Ryan Whalen (ed), *Computational Legal Studies* (Edward Elgar 2020); Jens Frankenreiter and Michael A Livermore, ‘Computational Methods in Legal Analysis’ (2020) 16 Annual Review of Law and Social Science 39. For discussion of the use of NLP in legal scholarship, including some use cases for Word2Vec, see Arthur Dyevre, ‘Text-Mining for Lawyers: How Machine Learning Techniques Can Advance our Understanding of Legal Discourse’ (2020) Erasmus Law Review (forthcoming, SSRN 3734430).

80 Tomas Mikolov and others, ‘Efficient Estimation of Word Representations in Vector Space’ (arXiv:1301.378, 2013).

81 Zellig Harris, ‘Distributional Structure’ (1954) 10 Word 146.

82 The two Word2Vec algorithms are ‘Skip-gram with negative sampling’ (SGNS) and ‘Continuous Bag of Words’ (CBOW). The first of these seeks to predict the immediate context of words around a target word, while the latter seeks to predict a target word from its immediate context. In both cases, the context window—that is, how many words around the target word—used for the prediction task is a hyperparameter set by the modeller.

83 Maria Antoniak and David Mimno, ‘Evaluating the Stability of Embedding-Based Word Similarities’ (2018) 6 Transactions of the Association for Computational Linguistics 107.

84 Mikolov and others (n 80) 2.

85 This example, vector(‘King’)—vector(‘Man’) + vector(‘Woman’), was first introduced by Mikolov and others, but it has since become part of the standard explanation for word embedding. Interestingly, Word2Vec manages to successfully encode syntactic relations as well (eg ‘apparent’ is to ‘apparently’ as ‘rapid’ is to ‘rapidly’). See ibid 5–9.

86 Emma Rodman, ‘A Timely Intervention: Tracking the Changing Meanings of Political Concepts with Word Vectors’ (2020) 28 Political Analysis 87.

87 Austin Kozlowski, Matt Taddy and James A Evans, ‘The Geometry of Culture: Analyzing the Meanings of Class through Word Embeddings’ (2019) 84 American Sociological Review 905.

88 Milan van Lange and Ralf Futselaar, ‘Debating Evil: Using Word Embeddings to Analyze Parliamentary Debates on War Criminals in the Netherlands’ (2018) Proceedings of the Conference on Language Technologies & Digital Humanities 147.

89 I implement Word2Vec with Python (a general-purpose programming language) using the Word2Vec module from the Gensim open-source Python library.

90 Griffith (n 14).

91 See Cornelia Ilie, ‘Strategic Uses of Parliamentary Forms of Address: The Case of the UK Parliament and the Swedish Riksdag’ (2010) 42 Journal of Pragmatics 885.

92 I wrote a web scraping programme in Python specifically for the purpose of extracting text from Hansard.

93 The complete list of these search terms, in alphabetical order, is: ‘British constitution’, ‘civil liberties’, ‘constitution’, ‘constitutional convention’, ‘constitution of the United Kingdom’, ‘devolution’, ‘fundamental rights’, ‘human rights’, ‘independence of the judiciary’, ‘judicial independence’, ‘judicial power’, ‘judicial review’, ‘ministerial accountability’, ‘parliamentary democracy’, ‘parliamentary sovereignty’, ‘parliamentary supremacy’, ‘prerogative power’, ‘representative democracy’, ‘royal prerogative’, ‘separation of powers’, ‘sovereignty of parliament’, ‘supremacy of parliament’, ‘the rule of law’, ‘United Kingdom’s constitution’ and ‘unwritten constitution’. Many of these web-scraping queries were at least partially redundant; because multiple search terms appear within the same debates, each round of web scraping does not necessarily add new debate texts to the corpus. However, to satisfy myself that the web-scraping process did not miss any relevant texts, I ran more queries than were strictly necessary.

94 Consistent with standard practice in word embedding, I did not remove so-called ‘stop words’ (eg ‘the’, ‘an’, ‘or’), nor did I stem or lemmatise words.

95 I used the *phrases* module from Gensim’s Python library, an algorithm that detects common phrases and *n*-gram collocations from a stream of sentences. See the documentation at ‘Phrase (collocation) detection’ (*Gensim: Topic Modelling for Humans*) <https://radimrehurek.com/gensim/models/phrases.html>.

96 ‘British constitution’ and ‘our constitution’ are by far the most common phrases used to refer to the UK’s constitution within parliamentary debate; in the Hansard corpus, ‘our constitution’ is used a total of 5751 times and ‘British constitution’ is used a total of 1478 times. I ignore atypical ways of referring to the UK’s constitution (eg ‘our constitutional system’ or ‘the United Kingdom’s constitutional arrangements’). Interestingly, ‘the constitution of the United Kingdom’ turns out to be a surprisingly rare phrase in the context of parliamentary debate; it appears only 204 times across the entire Hansard corpus and only 82 times in texts after 1997. The phrase ‘the United Kingdom’s constitution’ and ‘the UK’s constitution’ are even rarer; in Hansard’s transcripts (going back to 1800), the former appears only 37 times and the latter appears only 24 times.

97 See Loughlin, ‘In Search of the Constitution’ (n 47); Vernon Bogdanor, *The Coalition and the Constitution* (Bloomsbury Publishing 2011).

98 This approach should now be considered best practice, following experiments conducted by Antoniak and Mimno that found that training on 25 bootstrap samples, averaging similarity scores across models, stabilises the variance inherent in the machine learning process. See Antoniak and Mimno (n 83) 107.

99 The variability is inherent because Word2Vec algorithms begin with randomly assigned word vectors for every word or *n*-gram. The initial random vectors are altered over the course of many iterations of the machine learning process to optimise each vector. However, because the initial vectors are random, two models trained on the same corpus will generate slightly different word vectors and, consequently, there will be some variance in the vector cosine similarities across models trained on the same corpus.

100 See ibid. See also Arthur Spirling and Pedro L Rodriguez, ‘Word Embeddings: What Works, What Doesn’t, and How to Tell the Difference for Applied Research’ Journal of Politics (forthcoming).

101 See Antoniak and Mimno (n 83).

102 ibid.

103 Rodman (n 86) 96–7.

104 ibid.

105 This foundational model uses the SGNS Word2Vec algorithm, with 300 dimensions, five ‘epochs’ (ie machine learning cycles) and a context window of eight words (that is, eight words on either side of the target word). These hyperparameter settings are informed by recent experimental findings that improvements in model performance are marginal beyond 300 dimensions and a context window of six-to-eight words. See Spirling and Rodriguez (n 100). Training for the first sub-corpus is then initialised with the vectors from this foundational model and, thereafter, separate models are retrained for each sub-corpus in chronological order, using the vectors derived for each era to initialise the machine learning process for the next era.

106 Rodman (n 86) 105. For examples of the alternative approaches to diachronic word embeddings, see Robert Bamler and Stephan Mandt, ‘Dynamic Word Embeddings’ in *Proceedings of the 34th International Conference on Machine Learning* (ICML 2017) 380; William Hamilton, Jure Leskovec and Dan Jurafsky, ‘Cultural Shift or Linguistic Drift? Comparing Two Computational Measures of Semantic Change’ in *Proceedings of the 2016 Conference on Empirical Methods in Natural Language Processing* (Association for Computational Linguistics, 2016) 2116; Zijun Yao and others, ‘Dynamic Word Embeddings for Evolving Semantic Discovery’ in *WSDM 2018: The Eleventh ACM International Conference on Web Search and Data Mining* (Association for Computing Machinery, 2018); and Yoon Kim and others, ‘Temporal Analysis of Language through Neural Language Models’ in *Proceedings of the ACL 2014 Workshop on Language Technologies and Computational Social Science* (Association for Computational Linguistics, 2014) 61.

107 The results of these are reported in the Technical Appendix.

108
*p*-value < 0.001.

109 HC Deb 30 June 2015, vol 597, col 406WH (Alistair Carmichael MP). An earlier proposal for repealing the Act—the Human Rights Act 1998 (Repeal and Substitution) Bill—had been debated in Parliament, but this was a Private Member’s Bill promoted by Charlie Elphicke MP. See HC Deb 1 March 2013, vol 559, col 574.

110 HC Deb 30 June 2015, vol 597, col 424WH. On another occasion, Cherry refers to human rights as being ‘written into the devolution settlement by virtue of the Scotland Act 1998’. See HC Deb 21 October 2015, vol 600, col 1091. For other examples of this kind of claim about the repeal of the Human Rights Act, see HC Deb 2 March 2016, vol 606, col 342WH (Richard Arkless MP); HL Deb 2 July 2015, vol 762, col 2181 (Lord Wallace of Tankerness).

111 See feg HC Deb 24 January 2017, vol 620, col 157; HC Deb 8 September 2015, vol 599, col 219; HC Deb 30 June 2015, vol 597, col 409WH.

112 Walter Bagehot, *The English Constitution* (5th edn, Kegan Paul, Trench, & Co 1888) vii.

113 Bogdanor (n 1) 284.

114
*R (Miller) v The Prime Minister and Cherry v Advocate General for Scotland* [2019] UKSC 41.

115 For a qualitive analysis of how scholars of UK public law have used the term ‘constitutionalism’, see Jo Eric Khushal Murkens, ‘The Quest for Constitutionalism in UK Public Law Discourse’ (2009) 29 OJLS 427.

## Technical Appendix: Model Validation

Scepticism about new methods is healthy, especially where the methods in question rely on mathematical abstractions to measure qualities that cannot be observed directly. No measure is perfect—*we should not expect the map to be the territory*—but we can rightly ask for some assurances that the measures we use are reasonably valid, ie that they probably track the qualities we want them to track. In this spirit of healthy scepticism, this appendix canvasses some validity tests to demonstrate that the word-embedding models used in the article really do represent semantic relations between concepts.

A good place to begin validating a word-embedding model is to test how closely it tracks human judgments about semantics in general. People with a reasonable command of the English language will agree, for example, that the words ‘aeroplane’ and ‘jet’ are more semantically related to one another than either word is to the word ‘parliament’ or the word ‘courtesy’. They will also tend to agree about analogical relationships, for example that ‘woman’ is to ‘mother’ as ‘man’ is to ‘father’. The extent to which the semantic relationships encoded by a word-embedding model correlate with human judgments like these can be used to assess the model’s validity; we would expect that a good model would tend to agree with human judgments. Ideally, validation should be performed systematically, by testing the model against large datasets of the relevant sorts of human judgments.

For the purpose of assessing a model’s ability to measure the semantic similarity of words, a large dataset of word pairs and corresponding human judgments of similarity was developed (and is still maintained online) by Evgeniy Gabrilovich (a data scientist at Google).^116^ Applying the foundational Hansard debate model used in this article to this test collection yields positive and statistically significant correlations with human judgments: a Pearson’s correlation of 0.501 (*p*-value < 0.001) and a Spearman’s correlation of 0.528 (*p*-value < 0.001). These correlations are not perfect (a perfect correlation of ‘1’ would indicate that the model always agrees with the human judgments), but they are both statistically highly significant and large enough that they would generally be considered to show a moderate-to-strong association.^117^ Given that human judgments about semantic similarity do not correlate perfectly with one another either, these correlations should give us some confidence that the model provides a reasonably valid (if imperfect) measure of how semantically similar (or dissimilar) one concept is to another within a given corpus.

A similar systemic validation can be performed with analogies using the standardised accuracy test created by Mikolov and others’ team at Google.^118^ This test collection includes 8869 semantic questions based on analogies (eg ‘woman’ is to ‘mother’ as ‘man’ is to ‘father’), as well as 10,675 syntactic questions. The foundational Hansard model used in this article performs even better on this test—the model achieves a total accuracy score of 56% overall, which is slightly higher than the total score of 54% achieved by Mikolov and others’s similarly configured Word2Vec model (a model that was trained on a much larger corpus of 1.6 billion words).^119^ Although 56% may not sound incredible at first blush, one must bear in mind that the odds of the model arriving at the correct answer by random chance are extremely low; the model has to identify precisely the correct word (a synonym will not count), out of the model’s total vocabulary of 58,841 words and *n*-grams, to complete the analogy and score a point on the test.

Knowing that the foundational Hansard model does a respectable job of encoding static semantic relationships is encouraging, but the research purpose at hand requires that a chronological series of models trained on several sub-corpora can detect semantic changes across time. One way to check that the models can do this is to devise a validity test based on prior extrinsic knowledge about the world that created the corpus; once we can see that the models can detect a semantic change that we are nearly certain will have taken place, then we can have some confidence that the models can also detect semantic changes that we are less certain about.^120^

Given the nature of the corpus (ie parliamentary debate) and the way it has been divided for diachronic analysis (ie by turnover in political party control of government), an intuitive validity test of this kind is the extent to which the models detect changes in semantic relations that correspond to turnover in the office of Prime Minister. One would expect, for instance, that the word ‘Thatcher’ would become more semantically related to ‘Prime Minister’ during Margaret Thatcher’s tenure as Prime Minister; these two words—which are otherwise unrelated—abruptly (and for a defined period) share the same reference and, consequently, one would expect a corresponding change in vector cosine similarities. Likewise, during Tony Blair’s tenure as PM, we would expect the word ‘Blair’ to become more semantically related to ‘Prime Minister’.

Figure 6 shows the results of this validity test applied to the sub-corpora, plotting changing estimates of the semantic relatedness (as measured by vector cosine similarity) of the words ‘Thatcher’ and ‘Blair’ to the word ‘Prime Minister’ over time. The results do indeed confirm that the models can detect diachronic semantic change. The cosine similarity of ‘Thatcher’ to ‘Prime Minister’ jumps up (for a statistically significant difference) for the period of 1979–96 (Margaret Thatcher was PM for 11 of these years), then drops down again to its original level for the remaining time periods. Likewise, ‘Blair’ is estimated to become significantly more semantically related to ‘Prime Minister’ during the New Labour era (Tony Blair was PM for 10 of these years), but this estimate drops down again thereafter.^121^ In sum, the models appear to be sensitive enough to changing patterns of word use that they can detect semantic change over time.

